# Identification of prevalent leachate percolation of municipal solid waste landfill: a case study in India

**DOI:** 10.1038/s41598-024-58693-5

**Published:** 2024-04-17

**Authors:** Pervez Alam, Afzal Husain Khan, Raisul Islam, Ehab Sabi, Nadeem A. Khan, Tasneem Imtiyaz Zargar

**Affiliations:** 1 Department of Civil Engineering, Baba Ghulam Shah Badshah University, Jammu, Jammu and Kashmir India; 2 Civil Engineering Department, College of Engineering, Jazan University, P.O. Box. 706, 45142 Jazan, Saudi Arabia; 3 Department of Civil Engineering, GLA University, Mathura, UP India; 4 Interdisciplinary Research Center for Membranes and Water Security, King Fahd University of Petroleum and Minerals, 31261 Dhahran, Saudi Arabia

**Keywords:** Leachate, Municipal solid waste, Landfills, Groundwater, Temperature, Environmental chemistry, Environmental impact, Environmental sciences, Environmental social sciences

## Abstract

Landfill leachate forms when waste-inherent water and percolated rainfall transfer are highly toxic, corrosive, acidic, and full of environmental pollutants. The release of leachate from municipal solid waste (MSW) landfill sites poses a severe hazard to human health and aquatic life. This study examined the impact of leachate from Delhi’s Ghazipur landfill on the nearby groundwater quality. Analysis of leachate samples was done to determine various parameters such as total dissolved solids (TDS), hardness, alkalinity, electrical conductivity, pH, BOD_5_, COD, nitrate, sulphate, chloride and iron, and presence of coliform bacteria. Significant dissolved elements (22,690–34,525 mg/L) were observed in the samples, indicated by the high conductivity value (1156–1405 mho/cm). However, a stable pH range (6.90–7.80) of leachate samples was observed due to high alkalinity concentrations between 2123 and 3256 mg/L. The inverse distance weighing (IDW) interpolation tool from QGIS 3.22.7 developed spatial interpolated models for each parameter across the Ghazipur area. The IDW interpolated graphs of various parameters over the whole study area confirmed these contaminations. In addition, leachate and groundwater samples were physio-chemically analyzed, and temporal fluctuation in landfill waste has also been studied. The temporal fluctuation results showed that when heat is produced, transmitted, and lost throughout the waste system, the maximum temperature position fluctuates over time. The findings of this study highlight the critical importance of landfill management in reducing groundwater contamination from MSW leachate.

## Introduction

Municipal solid waste (MSW) encompasses a wide range of waste materials that originate from homes, businesses, institutions, and industries in urban areas^1^. It includes papers, plastics, glasses, metals, food scraps, clothes, yard wastes, and other miscellaneous waste materials^2^. The volume, composition, and management needs of the MSW make it an environmental, economic, and social challenge^3^. Reduced environmental effects of MSW may be achieved by analyzing energy generation and recycling possibilities from waste, which also has the added benefit of supplying a nearby source of electricity^4^. By 2025, it is projected that the yearly production of MSW will have increased by 2.2 billion metric tons due to urbanization, economic expansion, population growth, and changing lifestyles^5,6^.

Since most MSW is disposed of in open areas, many developing countries typically observe or engage in this behaviour. It ultimately causes the toxic leachate generated from the waste to contaminate nearby water bodies or percolate to reach groundwater. Leachate primarily results from solid waste, which occurs mainly through the process of percolation or leaching^7^. When precipitation or any other liquid encounters solid waste in a landfill or disposal site, it seeps through the various layers of waste, dissolving both suspended and dissolved contaminants along the way^8^. With high concentrations of inorganic ions, organic molecules, and other harmful substances, including heavy metals and ammonia, landfill leachate is a highly contaminated liquid^9^. Dissolved organic matter (DOM) in leachate can potentially interfere with microbial activity and foul membranes, impairing the coagulation phase’s efficiency^10^. A variety of treatments have been developed to treat landfill leachate, including biological treatment (such as activated sludge and fluidized bed reactor operations), chemical treatment (such as Fenton process and chemical precipitation), and physico-chemical treatment (such as adsorption and membrane processes).

Every waste landfill must have efficient leakproofing and drainage systems to reduce the environmental risk associated with landfill leachate^11^. The sealing system ensures leachate is not confined in the landfill while preventing rain from penetrating the landfill and releasing leachate to the environment. Leachate is moved to the basin via open or enclosed canal systems, where it can then be used in various landfill technology processes or processed in a wastewater treatment facility before being discharged into the sewer^12–16^. There is a severe risk of greatly exaggerating the emissions and environmental damage caused by landfills due to a lack of understanding of the dynamic nature of landfill leachate throughout its life cycle^17^. Although landfills have gained popularity as a low-cost and technically feasible solution for treating MSW, they are likely to substantially damage groundwater through solute leaching^18,19^.

A study conducted in China reported that open disposal of solid waste can have several adverse effects on the surrounding environment, public health, and aesthetics^20^. Further, the decomposing of organic matter produces methane gas and leachate, contributing to climate change and groundwater contamination^21^. According to Mor et al., leachate released or transported from waste can contaminate soil and water bodies, leading to ecosystem degradation^22^. Leachate remains a significant risk to groundwater even if hazardous waste is not dumped in municipal landfills^23,24^. Further, if leachate is not adequately contained or managed, it can flow into nearby surface water bodies such as rivers, lakes, or streams, causing pollution and endangering aquatic ecosystems^25^. It also emits foul odors and volatile organic compounds (VOCs) as it decomposes, contributing to air pollution in the surrounding area^26^.

Previous research has shown that the four major chemical groups comprising landfill leachate are DOM, inorganic compounds, heavy metals, and xenobiotic organic components^27^. Because acetogenic leachate contains more organic matter than methanogenic leachate, it has a higher BOD: COD ratio than the latter. The acetogenic leachate has higher concentrations of heavy metal contaminants due to its acidic composition, which increases metal solubility^28^. Due to changes in waste composition, water content, and seasonal variables like temperature and precipitation, landfill leachate properties show significant variability^29^. It has been observed from the literature survey that some researchers have carried out the effects of landfill leachate on groundwater^17,18,22,30,31^. However, our study navigates the intricate landscape of waste management, focusing on the pervasive issue of leachate percolation in Ghazipur landfill, India. With meticulous analysis and comprehensive data, we examine the environmental impact and implications of the prevalent leachate percolation in MSW landfills. Through this insightful case study, we unravel the environmental complexities, striving towards sustainable solutions for a cleaner, greener future. Thus, landfill leachate must be monitored to ensure human and environmental safety. Herein, this study investigated the leachate percolation through the soil into the underlying aquifers. The main objectives of this study are (a) to identify characteristics of leachate generated from landfill sites and (b) to identify the impacts on the groundwater quality using GIS-based interpolation techniques. To achieve these objectives, the inclusion of field measurements, GPS data, and physico-chemical parameters was assessed. Furthermore, an evaluation of the temporal evolution with depth and time was also made on the landfill’s site.

## Methodology

The study area, sample collection and preparation, temporal variation, inverse distance weighting interpolation technique, and water quality index will be discussed in the subsequent sections of the methodology.

### Study area

The current research was carried out at the Ghazipur landfill in New Delhi, which has been in operation since 1984 and overflowing since 2002. However, waste has still been put there despite the landfill’s capacity being surpassed for at least 10 years. The landfill had surpassed 65 m at the most recent count in 2019 (213 feet). The latitude and longitude of the Ghazipur landfill site are 28° 37′ 27.2064′′ N and 77° 19′ 37.8372″ E, respectively. Delhi City generates around 11,144 tons of MSW daily, deposited at open dumpsites at different landfill sites. Among all landfill sites, the major portion of MSW over the last two decades has been diverted to the Ghazipur landfill. Therefore, several fire and smouldering accidents were observed at the Ghazipur landfill, which affected those residing in the nearby housing societies, slums, and schools who complained of difficulty breathing, itching in the eyes, etc.

Further, the study area has also been selected due to its significance as one of the largest and most prominent waste disposal sites in the region. The environmental challenges it poses make it interesting for research. The landfill reflects the acute waste management issues facing the city and raises questions about the impacts of rapid urbanization, population growth, and unsustainable consumption patterns on the environment and public health.

### Sample collection and preparation

#### Chemical composition of leachate

The original MSW content, the level of compaction, the site’s hydrology, the climate, and lastly, landfill age all affect the chemical components of leachate. For these reasons, there is significant variation in the leachate characteristics produced from landfills. Ghazipur landfill site is an open dumping site with no liners, leachate collection system, or arrangement for gas collection. Hence, leachate production rate and characteristics are more complex than sanitary landfills. The monthly collection of six leachate samples was done between May 2021 and October 2021 from the landfill of Ghazipur. A comprehensive total of 36 samples were systematically collected across the study area. This sampling protocol was determined based on an initial assessment, which concluded that six strategically positioned samples adequately represented the entire landfill area. These selected sampling locations were confirmed to encompass all classes of waste present in the landfill. The sampling was meticulously done to ensure that the leachate collected was produced from all types of waste in the landfill, thereby maintaining homogeneity throughout the sampling process.

Leachate sampling was done as follows and outlined by Cerne and Junestedt^32^. Leachate sampling was done using grab sampling in 1000 mL bottles made from plastic cleaned thoroughly, and the samples were then stored at 4 °C. The laboratory analyzed various samples using standardized methods outlined in the American Public Health Association’s (APHA) Standard Methods for the Examination of Water and Wastewater^33^. The parameters examined included pH, electrical conductivity, BOD_5_, COD, hardness, alkalinity, total dissolved solid (TDS), chloride, sulphate, nitrate, and iron. In addition to the above, the samples have been tested for the presence of coliform bacteria because the coliform group of bacteria is the principal indicator of the suitability of water. Further, the following precautions have been taken during the collection of samples^34^.

- Thoroughly clean containers with laboratory detergent rinse and deionized water were used to collect samples.
- The samples are properly handled to avoid contamination. The sample was stored in dark, cold conditions, adjusted to 4 °C within 6 h, and promptly delivered to the laboratory.
- Care has been taken to avoid touching container openings to prevent contamination.
- Collected samples has been directly transferred into clean bottles to prevent contamination.

#### Analysis of groundwater samples in the proximity of Ghazipur landfill

**Figure 1:**
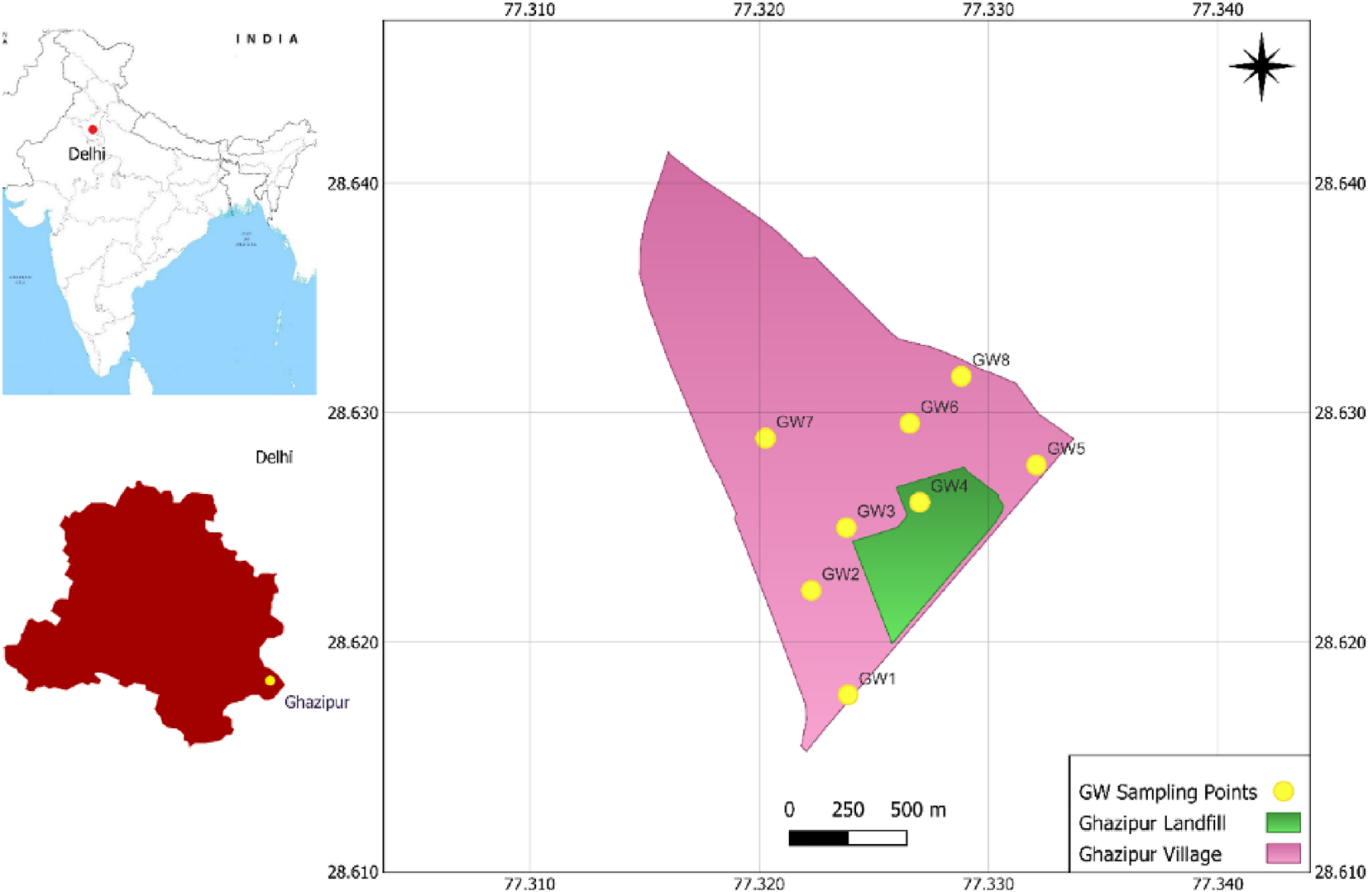
Location of study area on the Map of India and various ground water sampling points.

In order to better understand how landfill leachate affects groundwater, samples from hand pumps already in place at the Ghazipur landfill site are being taken. From May 2021 to October 2021, six groundwater samples from each area were taken monthly. Clean plastic bottles were used for a 1000 mL grab sampling, and samples were subsequently held at 4 °C in the environmental laboratory. The laboratory analyzed samples for various parameters following the American Public Health Association (APHA), Standard Methods for the Examination of Water and Wastewater (1998). The parameters examined included iron, pH, electrical conductivity, BOD_5_, COD, hardness, alkalinity, chloride, sulphate, nitrate, and total dissolved solids (TDS). In addition to the aforementioned, the samples have been examined for coliform bacteria. The cornerstone for bacteriological water quality standards has been the density of coliform group bacteria, which measures the level of contamination. Although it would be ideal if all samples were free of coliform bacteria, the reality is that no water sample should include more than 10 coliform bacteria per 100 mL. Table 1 lists the locations of groundwater test points, depth and their separation from the landfill site, and Fig. 1 illustrates the same information visually.

**Table 1:** Locations of groundwater samples (HP: hand pump).

Sample number	Source of collection	Depth of sample collection (m)	Coordinates		Distance from landfill (m)
			Latitude (°N)	Longitude (°E)	
GW1	HP	24	28.61771	77.32388	220 (West)
GW2	HP	30	28.62226	77.32227	235 (South-West)
GW3	HP	30	28.625	77.3238	190 (North-West)
GW4	HP	31	28.6261	77.327	25 (South)
GW5	HP	25	28.62772	77.33209	220 (East)
GW6	HP	26	28.62953	77.32657	290 (North)
GW7	HP	23	28.62888	77.32028	375 (North-West)
GW8	HP	29	28.63158	77.32881	380 (North-East)

### Temporal variation

Thermocouples, for example, have been used to detect the temperature in landfills with a wide range of other sensors^35,36^ and thermistors, e.g.^37^. According to the researchers, type K thermocouples are excellent for landfill applications because of their exceptional resilience to chemical conditions^38^.

Temperatures were recorded at several points in the landfills, such as the waste mass, cover, and peripheral control areas. Type K thermocouples were placed in specially made-arrays to measure temperatures^39^. Due to the rugged temperature measuring device usage, Type-K thermocouple was preferred over any other type of device. In addition to that, due to its broad temperature range, longer lifespan, prompt reaction, affordability, reliability and compact nature, this temperature measuring device was preferred.

For horizontal installations buried in trenches underneath liners, coverings, and wastes, the arrays spanned from 150 to 250 m in length. Vertical installations between 1 and 50 m high were built in boreholes through waste and coverings and at control sites. Following waste placement, vertical installations were created that made it possible to measure temperature changes with depth and waste age at a specific spot. During waste placement or liner/cover construction, horizontal installations were installed that made it possible to determine temperatures at a spot with a single waste age and a particular depth. All measurements were taken on a weekly basis.

### Inverse distance weighting interpolation technique

Inverse distance weighting (IDW) is a commonly used technique in spatial interpolation, which is a method used to estimate values at unsampled locations within a defined area based on measured values at sampled locations. We have selected this approach because our study involves spatially distributed data points with a relatively high density. Further, it is suitable for interpolating values from nearby data points, making it well-suited for datasets with dense spatial coverage. In addition, unlike other interpolation techniques, such as kriging, IDW does not require assumptions about the underlying distribution of the data. This flexibility is advantageous when dealing with environmental datasets where the data distribution may be complex or unknown. Given these considerations, IDW is the preferred interpolation technique for our study. While other interpolation methods may offer advantages in specific scenarios, IDW aligns well with the characteristics of our data and the objectives of our analysis, making it the most suitable choice for estimating values across the study area.

### Methodology for water quality index

The methodology employed for calculating the water quality index (WQI) in this research encompasses a systematic approach involving several essential steps and mathematical formulas. The observed values were compared with established standard values as per guidelines provided by BIS 10500. Weighted Normalized Scores (W_n_) for each parameter were computed utilizing the formula:

$${W}_{n}=\frac{k}{S},$$

where k represents the reciprocal of the standard value of each parameter, and S denotes the standard values. Subsequently, Q_n_ for each parameter were derived by the formulae:

$${Q}_{n}=100\times \frac{O-I}{S-I},$$

where O represents the observed value, I represent the ideal value (7 for pH, 0 otherwise), and S representing the standard value of the parameter. The weighted sum (W_n_ Log Q_n_) was then calculated and finally summed up. Finally, the WQI was computed by:

$$WQI=\frac{\sum {W}_{n}{Q}_{n}}{\sum {W}_{n}}.$$

## Results and discussion

The obtained results of leachate characteristics, effect on groundwater, temporal variation in landfill and water quality index has been discussed in the subsequent sections of result and discussion.

### Leachate characteristics

The pH values of the leachate samples ranged from 6.9 to 7.8 due to high alkalinity concentrations between 2123 and 3256 mg/L. The pH of leachate collected from an emerging and young landfill is usually less than 7, due to the production of carboxylic acids. However, as time passes, the pH of leachate usually turns from acidic to alkaline due to the formation of alkaline compounds, which indicates anaerobic biodegradation and a methanogenic stage of decomposition^40^. The pH values obtained from the leachate indicate the dominance of alkaline compounds and hence point out the maturity of the landfill.

The high conductivity value (1156–1405 mho/cm) represents the presence of salts in the samples (22,690–34,525 mg/L). The dissolved material concentration and conductivity are high for active landfills, whereas the conductivity values for abandoned landfills are often lower^41^. Leachate sample hardness ranged from 4312 to 5925 mg/L. The hardness was found to be high during the wet period of the year (July and August).

The chloride concentration was highest in October (2300 mg/L) and lowest in August (1760 mg/L). Because it is neither physically nor physiologically reactive, it is abundant and largely not preserved by soil systems. It spreads swiftly and frequently, indicating the progress of a plume of tainted water. Such large deviation in the chloride content could be linked to the precipitation, which may cause significant leaching of pollutants. The range of chloride content of active landfill leachate was reported by^41^ as 853 mg/L to 2670 mg/L. BOD_5_ and COD values were found high, of the order 2216 mg/L and 7998 mg/L, respectively, indicates severe contamination and may lead to direct groundwater pollution.

**Figure 2:**
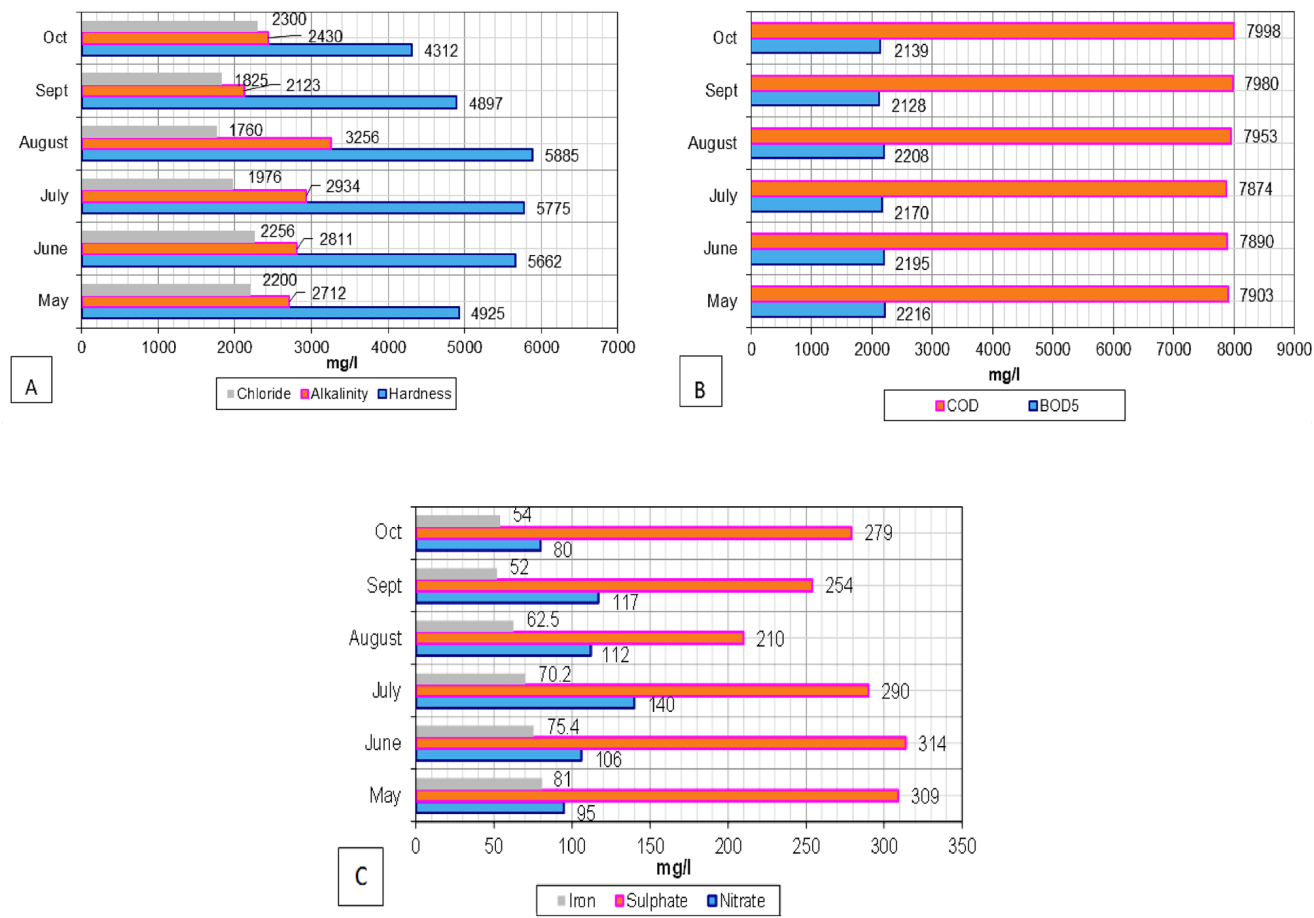
Variation of leachate characteristics in Ghazipur landfill (**A**) hardness, alkalinity, and chloride, (**B**) BOD_5_ and COD, and (**C**) nitrate, sulphate and iron.

Furthermore, it is worth pointing out that the values of BOD_5_ and COD almost remained stable throughout the analysis. This may be attributed to the stabilized chemical reactions in the landfill; on the contrary^42^ obtained fluctuations in the COD values of leachate collected from active and un-stabilized landfills. The BOD_5_/ COD value calculated for maximum values of BOD_5_ and COD was 0.27, which lies in the biodegradable zone^43^. Figure 2A,B illustrate the changes in hardness, alkalinity, chloride, BOD_5_, and COD. The nitrate concentration was highest (140 mg/L) in July and lowest (80 mg/L) in October. Sulphate values ranged from 210 to 309 mg/L, and iron values were very high (54–81 mg/L). It suggested that steel and iron are also being disposed of in landfills, where they might cause groundwater to become reddish-brown.

Micronutrients that are necessary for the growth of plants include metals like Fe and Ni. As a result, at some concentrations, they are required and can promote growth, but once they reach specific levels, they become poisonous to them. Metals in excessive amounts can interrupt germination and impede the growth of roots or shoots^44,45^. The concentrations of sulphate, nitrate and iron are shown in Fig. 2C. A comparison is drawn between the concentration of contaminants in leachate and municipal wastewater samples, as shown in Table 2. It could be seen that the Ghazipur landfill leachate concentration is very high on comparing various contaminant concentrations of typical wastewater and indicates heavy pollution of surface water and groundwater. Due to the high biodegradable content of MSW in Delhi, it is evident that it will produce leachate with more organics. Some hazardous, industrial, and hospital wastes are disposed of at the MSW disposal site. As a result, the leachate has substantially greater levels of pollution.

**Table 2:** Physico-chemical parameter concentrations of typical wastewater and leachate sample from Ghazipur landfill site.

Parameter (s)	Average value (s)	Range	Typical wastewater concentration
pH	7.4	6.9–7.8	6–8.5
Electrical conductivity (mho/cm)	1314	1156–1405	–
BOD_5_ (mg/L)	2176	2128–2216	–
COD (mg/L)	7933	7874–7998	250–1000
Hardness (mg/L)	5002	4312–5623	–
Alkalinity (mg/L)	2711	2123–3256	50–200
TDS (mg/L)	28,509	22,690–34,525	250–850
Chloride (mg/L)	2041	1765–2294	30–100
Sulphate (mg/L)	276	210–314	–
Nitrate (mg/L)	107	76–136	0
Iron (mg/L)	65.95	52–78.6	0.05–0.1
No. of coliform (MPN/100 mL)	262	240–300	–

The pollution index devised by Kumar and Alappat^46^, serves as a practical tool for evaluating the potential contamination posed by leachate discharged from municipal solid waste (MSW) landfills. Leachate from landfills can contaminate nearby soil and water sources, necessitating a method to quantify this risk. The index fulfills this need, aiding in identifying landfill sites requiring immediate attention. The leachate pollution index (LPI) can be calculated using the equation:

$$LPI={\sum }_{i=1}^{n}\left({w}_{i} {p}_{i}\right),$$

where LPI—the weighted additive leachate pollution index, w_i_—the weight for the ith pollutant variable, p_i_—the sub index value of the ith leachate pollutant variable, n—number of leachate pollutant variables used in calculating LPI and $\sum {w}_{i}=1$^47^.

The overall weights and subindex values considered in this study were taken in accordance with previous studies^47,48^. Now, overall LPI,

$$LPI=0.232 {LPI}_{org}+0.257 {LPI}_{inorg}+0.511{LPI}_{heavy \,metals}.$$

In this study, LPI value of 23.7 is found using the above calculated values, which is in line with the previous studies.

### Effect of leachate on groundwater

**Figure 3:**
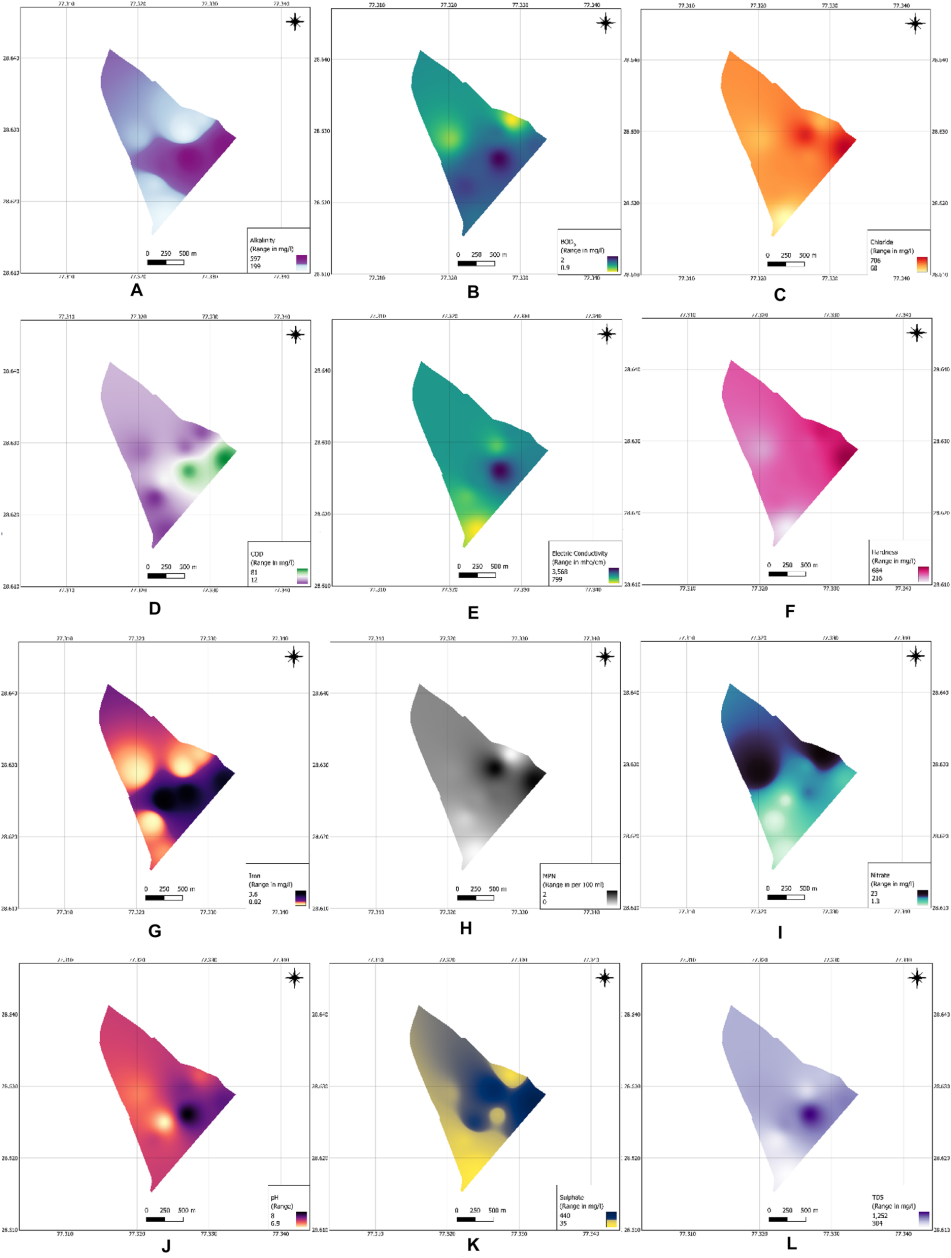
IDW Interpolated graphs of various parameters over the whole study area (**A**) alkalinity, (**B**) BOD_5_, (**C**) chloride, (**D**) COD, (**E**) electric conductivity, (**F**) hardness, (**G**) iron, (**H**) MPN, (**I**) nitrate, (**J**) pH, (**K**) sulphate, (**L**) TDS.

The results of the present investigation are summarized in Table 3, which provides a comprehensive picture of the characteristics of groundwater in and around the Ghazipur landfill site. The mean value of all the parameters was interpolated over the whole Ghazipur village and intensity models were prepared using Inverse Distance Weighing (IDW). Such models were trained using QGIS 3.22.7, as shown in Fig. 3A–L. In this investigation, parameter values were predicted using the geo-statistical modelling tool (QGIS 3.22.7) by averaging the parameters of sample data points near each known data point. The sample point map and the data at each sample point were imported into the QGIS software, and the IDW technique was used to interpolate the digitized values at unmeasured places. The average deviation between the neighbouring data and the un-sampled regions was measured experimentally using the IDW approach.

**Table 3:** Leachate pollution index of Ghazipur landfill.

Parameter (s)	Average value (s)	Weight (w)	Subindex value
LPI organic			
BOD_5_ (mg/L)	2176	0.158	62
COD (mg/L)	7933	0.322	24
No. of coliform (MPN/100 mL)	262	0.52	100
Summation		1	69.524
LPI inorganic			
pH	7.4	0.214	15
TDS (mg/L)	28,509	0.195	63
Chloride (mg/L)	2041	0.3546	10
Nitrate (mg/L)	107	0.2364	2
Summation		1	19.5138
LPI heavy metals			
Iron (mg/L)	65.95	1	5
Summation		1	5

The core concept of IDW (Inverse Distance Weighting) interpolation involves utilizing a collection of sample points in a weighted linear combination. This method relies on statistical and mathematical techniques to construct surfaces and predict locations where measurements are unavailable^30^.

**Figure 4:**
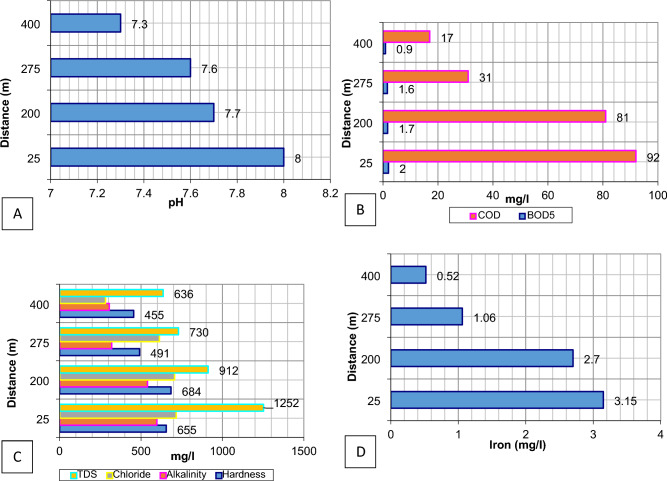
Variations of physico-chemical parameters with respect to distance from the landfill (**A**) pH value, (**B**) BOD_5_ and COD value, (**C**) TDS, chloride, alkalinity and hardness value, and (**D**) iron concentration.

The impact of leachate on groundwater quality parameters with respect to the distance from the landfill site is shown in Fig. 4A–D. Using the interpolated spatial models, the regions of groundwater affected by the landfill leachate can be easily determined. As seen in the spatial models, the parameters had greater values inside and at the boundaries of the landfill site, which seemed to decrease as the distance from the landfill site increased. The concerned authorities can use these interpolated models to monitor and improve the groundwater at critical points, whose location can be easily determined by the interpolated spatial models.

The pH value in the wells near the landfill (GW4) is more than that far from the landfill (GW8), which indicates that the water is more alkaline near the landfill, as shown in Fig. 4A. Groundwater samples showed a wide conductivity range from 799 to 3568 mho/cm. As shown in Table 4, the conductivity of groundwater is low and within the desired value in the South-West of the landfill (GW1) and in the west (GW2), whereas high conductivity of water at the North of the landfill and in the points near the landfill (GW4 and GW5). Such typical pH and Electrical Conductivity values indicate leaching leachate into the groundwater. The alkaline nature of water and high conductivity values near the landfill suggest a severe risk of groundwater pollution^31^. The BOD_5_ and COD values for all wells are more than the desirable limit, indicating severe groundwater contamination around the landfill, especially in the points near the landfill (GW4, GW5 and GW6), as shown in Fig. 4B. The hardness, alkalinity, total dissolved solids (TDS), and chloride were high and more than the desirable limit in all groundwater wells except for GW1, located in the South-West of the landfill. These values were very high in the wells near the landfill (GW4 and GW5), which is more than that far from the landfill (GW8), as shown in Fig. 4C. Sulphate values are within the desirable limit in the South-West landfill (GW1) and the west landfill (GW2).

**Table 4:** Physico-chemical characteristics of groundwater near Ghazipur landfill site.

Location	No. of samples	Min	Max	Average	St. D	% Error at 95% confidence
pH						
GW1	6	6.9	7.8	7.4	0.33	0.22
GW2	6	7	7.8	7.4	0.31	0.2
GW3	6	6.7	7.2	6.9	0.21	0.14
GW4	6	7.7	8.3	8	0.24	0.16
GW5	6	7.1	8.3	7.7	0.4	0.26
GW6	6	7.2	7.9	7.6	0.24	0.16
GW7	6	6.8	7.6	7.2	0.29	0.19
GW8	6	7.1	7.5	7.3	0.18	0.12
Conductivity (mho/cm)						
GW1	6	775	817	799	15.94	10.41
GW2	6	1491	1508	1499	6.05	3.95
GW3	6	2244	2268	2255	9.25	6.04
GW4	6	3557	3580	3568	8.37	5.47
GW5	6	2342	2362	2354	7.13	4.66
GW6	6	1509	1526	1517	5.66	3.7
GW7	6	2103	2118	2110	5.4	3.53
GW8	6	2251	2262	2256	4	2.61
BOD_5_ (mg/L)						
GW1	6	1.2	2	1.6	0.32	0.21
GW2	6	1.5	2.2	1.8	0.24	0.16
GW3	6	1.5	2	1.7	0.18	0.12
GW4	6	1.8	2.2	2	0.14	0.09
GW5	6	1.4	2.1	1.7	0.26	0.17
GW6	6	1.9	2.3	1.6	0.14	0.09
GW7	6	0.8	1.3	1.1	0.21	0.14
GW8	6	0.8	1.1	0.9	0.13	0.08
COD (mg/L)						
GW1	6	12	16	14	1.41	0.92
GW2	6	10	14	12	1.41	0.92
GW3	6	42	54	47	4.38	2.86
GW4	6	84	98	92	5.48	3.58
GW5	6	77	85	81	3.52	2.3
GW6	6	28	35	31	2.61	1.71
GW7	6	19	25	22	2.28	1.49
GW8	6	14	21	17	2.61	1.71
Hardness (mg/L)						
GW1	6	209	221	216	4.15	2.71
GW2	6	458	465	462	2.76	1.8
GW3	6	465	480	473	6.2	4.05
GW4	6	649	662	655	4.43	2.89
GW5	6	674	692	684	7.67	5.01
GW6	6	483	497	491	5.18	3.38
GW7	6	352	373	363	8.56	5.59
GW8	6	446	462	455	6.26	4.09
Alkalinity (mg/L)						
GW1	6	187	209	199	8.39	5.48
GW2	6	297	324	313	9.08	5.93
GW3	6	408	426	418	5.8	3.79
GW4	6	587	606	597	6.51	4.25
GW5	6	526	556	540	10.26	6.7
GW6	6	312	325	320	4.82	3.15
GW7	6	336	352	344	6.23	4.07
GW8	6	289	315	305	9.32	6.09
TDS (mg/L)						
GW1	6	295	311	304	6.54	4.27
GW2	6	401	426	414	10.58	6.91
GW3	6	828	860	844	12.51	8.17
GW4	6	1238	1266	1252	11.45	7.48
GW5	6	892	924	912	11.82	7.72
GW6	6	720	739	730	6.78	4.43
GW7	6	743	772	757	11.1	7.25
GW8	6	624	645	636	8.12	5.3
Chloride (mg/L)						
GW1	6	54	76	68	8.02	5.24
GW2	6	347	370	360	8.2	5.36
GW3	6	231	423	385	75.68	49.44
GW4	6	709	724	716	5.4	3.53
GW5	6	680	722	706	14.75	9.64
GW6	6	595	626	613	10.32	6.74
GW7	6	255	278	268	9.14	5.97
GW8	6	274	289	282	5.93	3.87
Sulphate (mg/L)						
GW1	6	26	40	35	5.18	3.38
GW2	6	148	161	155	4.47	2.92
GW3	6	259	275	266	5.93	3.87
GW4	6	212	226	219	5.55	3.63
GW5	6	432	446	440	5.51	3.6
GW6	6	312	337	325	8.94	5.84
GW7	6	220	236	229	5.66	3.7
GW8	6	132	141	136	3.35	2.19
Nitrate (mg/L)						
GW1	6	1.1	2.1	1.6	0.4	0.26
GW2	6	0.9	1.7	1.3	0.32	0.21
GW3	6	1.7	2.3	2	0.24	0.16
GW4	6	11.9	14	12.8	0.89	0.58
GW5	6	6.6	7.3	7	0.26	0.17
GW6	6	10.7	11.1	10.9	0.15	0.1
GW7	6	17	25	21	3.22	2.1
GW8	6	19	27	23	3.22	2.1
Iron (mg/L)						
GW1	6	0.75	0.81	0.78	0.03	0.02
GW2	6	0	0.04	0.02	0.01	0.01
GW3	6	3.3	3.9	3.6	0.24	0.16
GW4	6	3.1	3.2	3.15	0.04	0.03
GW5	6	2.65	2.75	2.7	0.03	0.02
GW6	6	1.01	1.11	1.06	0.04	0.03
GW7	6	0.15	0.19	0.17	0.02	0.01
GW8	6	0.49	0.55	0.52	0.02	0.01
No. of coliform MPN/100 mL						
GW1	6	0	0	0	0	0
GW2	6	0	1	0.33	0.52	0.34
GW3	6	0	2	0.83	0.98	0.64
GW4	6	0	3	1.17	1.17	0.76
GW5	6	0	5	2	2.1	1.37
GW6	6	0	4	2	1.9	1.24
GW7	6	0	2	0.83	0.98	0.64
GW8	6	0	0	0	0	0

In contrast, it is high in the other wells. Nitrate value and the number of coliform bacteria are low for all wells, indicating no bacteriological contamination in groundwater. Iron value was high in all wells, especially in the wells near the landfill (GW4, GW5 and GW6), except for the wells GW2, and GW7, as shown in Fig. 4D. It could be seen from the physio-chemical characteristics of groundwater (Table 2) that the groundwater quality around the Ghazipur landfill site surpassed the desired criteria, and it does not meet the drinking water level. The groundwater contamination is generally worse in landfills in the North and North-Western regions. The areas close to the dump have higher pollution levels. It steadily decreases as it gets farther away towards the north and west, indicating that the landfill leachate is having a negative impact on the groundwater in the landfill and that the groundwater is flowing in a North-Western direction. Further, as one moves away from the dump towards the North and West, pollution levels gradually decrease, suggesting a downstream flow direction of groundwater.

### Temporal variation in landfill

The biological decomposition of waste in landfills releases gases, heat, and leachate as a by-product. Various up-to-date technologies are available to collect methane gas released from landfills, but it’s very problematic to utilize a large amount of heat generated from landfills due to the exothermic reaction inside the landfills. Because the right temperature is crucial for the continuous biological decomposition processes and methane generation, it is imperative to prevent undercooling. A temperature of 35 to 40 °C and 50 to 60 °C were the ideal temperature range for developing mesophilic and thermophilic bacteria engaged in garbage decomposition. The ideal temperature range for gas generation at a landfill was determined to be between 40 and 45 °C. The thermal regime of MSW landfills was thoroughly examined in this work, including the variations in temperature with depth and waste age.

**Figure 5:**
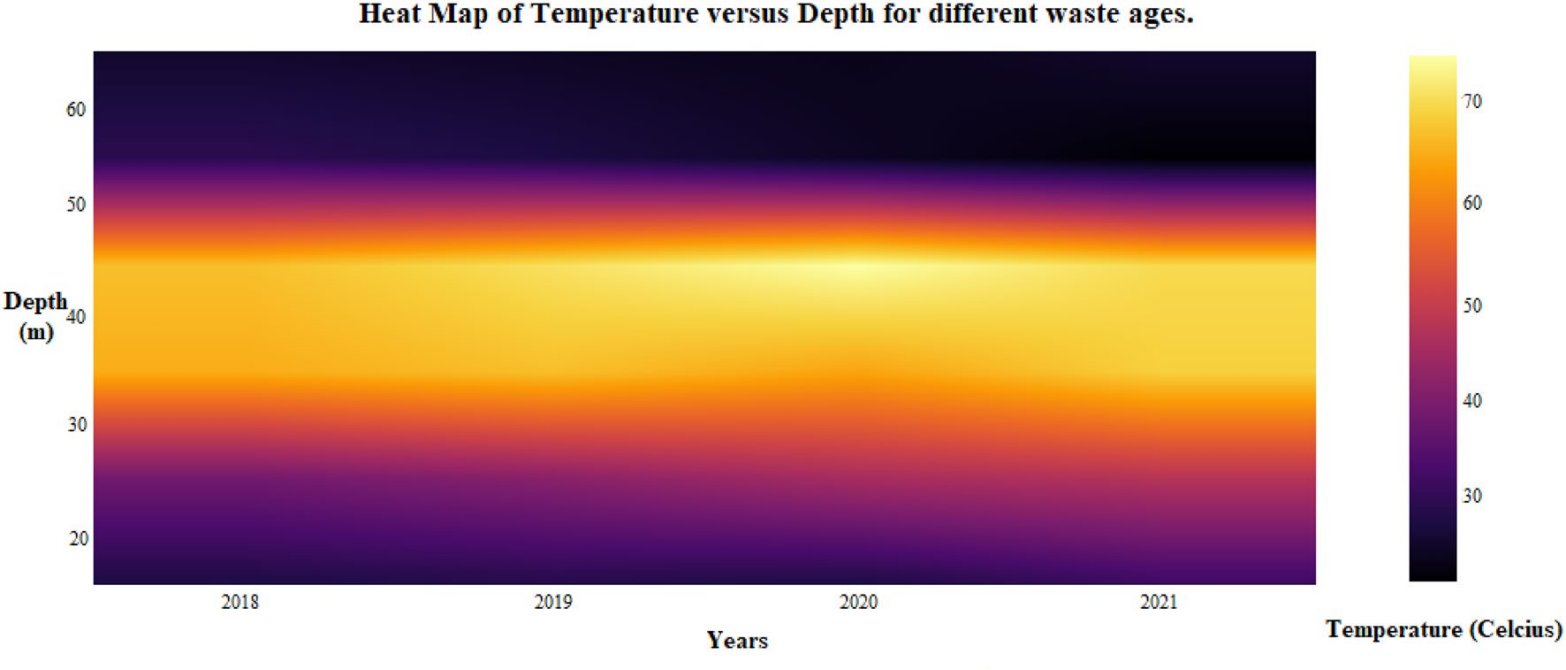
Temperature versus depth profile at different waste age.

The temperature data were gathered from the Ghazipur landfill to explore the typical temperature profile with respect to depth. Temperature versus depth comparisons between years were done at this location. Low temperatures were recorded over and under this intermediate zone of a landfill, while maximum temperatures were recorded close to its mid-depths. The position of the highest temperature changes over time as heat is produced, distributed, and dissipated across the landfill system. The average temperature variation for this landfill with depth is presented in Fig. 5, where waste was dumped from 1989 to 2019. The depiction in Fig. 5 shows that waste temperature increases as time passes, and the heat zone continuously changes with depth. The landfill subsoil could be broadly categorized into three zones based on the magnitude of temperature. Zone-1 can be established upto the depth of 30 m from the surface and has a temperature range of 30–50 °C. Zone-2 is identified to start at a depth of 30 m and end at 50 m from the surface. The temperature of this zone is the maximum of all and lies between 60 and 70 °C. Zone-3 extends beyond Zone-2 up to a depth of 60 m. This zone is the coolest, and the temperature is below 30 °C. Similar observations were made by^49^, i.e., the highest temperatures were recorded at central spots in the middle third of the waste mass depth.

Municipal solid waste (MSW) landfills operate at 20–65 °C, usually below 55 °C^49^. MSW landfills have had temperatures above 80 to 100 °C during the previous 10 years^50,51^. Such landfills that exhibit higher temperatures than usual are called elevated temperature landfills (ETLs). The Ghazipur landfill can be accepted as an ETL based on the temperature data obtained. The gases produced in such landfills may be devoid of methane and may lead to the release of more odorous compounds. Furthermore, the liquid pressure may increase due to high temperatures and lead to unexpected leachate outbreaks^50,52^.

The biological, chemical, and geomechanical reactions in waste liners and coverings are influenced by temperature and heat transport^49^. Laboratory and field investigations have indicated that the ideal temperature ranges for gas generation from waste degradation are between 34 and 45 °C. At temperatures between about 20 and 75 °C, much lower gas production rates are anticipated. Temperature has an impact on the engineering characteristics of wastes; for instance, in laboratory studies, waste compressibility rises by almost double as the temperature increases from 20 to 35 °C. Elevated temperatures can have detrimental effects on lining systems, leading to various negative outcomes. Geosynthetic materials used for lining landfills or containment structures experience a reduced lifespan under high temperatures. The increased heat can cause accelerated degradation, compromising their integrity and performance over time^53^. However, using thermal and biochemical transformations to produce energy-rich substrates and gases for heat and electricity production are also reported^33,54,55^.

### Water quality index

The detailed calculation of the water quality index using BIS standards is shown in Table 5. It has been observed from the calculation that the WQI is found to be 537.3, indicating very poor quality, as reported by Chidiac et al.^11^ and not fit for drinking. This comprehensive index furnishes a singular numerical representation signifying the overall water quality status, thereby facilitating effective communication of research findings to pertinent stakeholders and decision-makers for water resource management strategies^34,38,39^.

**Table 5:** Water quality index using BIS standards.

Parameter	Standard value	(Standard values)^−1^	Observed value	W_n_	Q_n_	W_n_Q_n_
pH	7.5	0.1	7.4	0.1	87.5	8.75
Conductivity (mho/cm)	300	0.0	2044.8	0.0	681.6	0
COD (mg/L)	4	0.3	39.5	0.1	987.5	98.7
Hardness (mg/L)	200	0.0	474.9	0.0	237.4	0
Alkalinity (mg/L)	200	0.0	379.5	0.0	189.8	0
TDS (mg/L)	500	0.0	731.1	0.0	146.2	0
Chloride (mg/L)	250	0.0	424.8	0.0	169.9	0
Sulphate (mg/L)	200	0.0	225.6	0.0	112.8	0
Nitrate (mg/L)	45	0.0	10.0	0.0	22.1	0
Summation		0.4				107.45

### Control of leachate generation-related groundwater contamination

Controlling leachate to control groundwater pollution requires a holistic approach that combines monitoring and preventive measures. Implementing leachate collection devices, maintaining landfills properly, and enforcing strict rules on waste disposal practices are all part of prevention. In order to identify contamination early on, monitoring is comprised of routinely evaluating groundwater quality by sample and analysis. Groundwater quality and pollution can be improved by the employment of remediation solutions such as permeable reactive barriers, natural attenuation techniques, and pump-and-treat systems. Furthermore, it is critical to utilize community involvement and public awareness initiatives to promote sustainable waste management procedures and an environmental protection policy in order to maintain groundwater resources for future generations.

## Future prospects

The Ghazipur landfill is surrounded by the Hindon-Yamuna canal on two sides, ultimately falling into the Yamuna River. Due to the Ghazipur landfill, the subsurface water pollution will ultimately pollute or may be currently polluting one of the major rivers of India. Thus, the scope of this work may be further widened by developing a method to reduce subsurface pollution by incorporating various geosynthetic, clay or composite liner systems.

Furthermore, the water quality of the major surface water bodies surrounding the Ghazipur landfill site may be studied, and the effect of the subsurface water pollution, as reported in this study, can also be found. In addition, the concerned authorities must take note of the deteriorating underground water quality, and efficient water treatment plans must be designed to ensure a safe livelihood. The temporal variations of the landfill are another factor to be considered in compliance. The zone of maximum temperature continuously changes its dimensions (increasing), which is not suitable for the life of a landfill. The composition of the input waste must be carefully decided so that the temperature fluctuations are normalized. Another aspect to be taken care of is the increasing height of the landfill. However, utilizing existing processes to maintain the landfill height by integrating geo-reinforcements is necessary to assure stability and safety.

## Conclusion

Leachate and groundwater samples were collected near the MSW Ghazipur landfill in Delhi and selected based on predetermined sampling points and distance from the site. Laboratory tests were conducted to assess their physico-chemical properties and parameters, following guidelines from the American Public Health Association’s (APHA) Standard Methods for the Examination of Water and Wastewater. Leachate samples exhibited pH values ranging from 6.9 to 7.8, with significant alkalinity concentrations (2123–3256 mg/L) and high conductivity (1156–1405 mho/cm), indicative of dissolved particle presence. Leachate hardness ranged from 4312 to 5925 mg/L, while chloride concentrations varied from 176 to 2300 mg/L. Groundwater pH levels were higher closer to the landfill, with conductivity ranging from 799 to 3568 mho/cm. Most groundwater wells exceeded the desired limits for total dissolved solids (TDS), chloride, hardness, and alkalinity, particularly in northern and north-western areas. Leachate from the landfill significantly influenced groundwater flow, decreasing steadily with distance north and westward, as demonstrated by spatial interpolation models. Temporal analysis revealed the landfill’s classification as an elevated temperature landfill (ETL), with the highest temperature zone widening over time and moving towards the surface, raising concerns. However, no significant changes were observed in the width of zone 3. Apart from these conclusions, the study has some limitations, including overlooking long-term ecosystem and human health impacts, socioeconomic implications for nearby communities, and alternative waste management strategies. Addressing these gaps requires interdisciplinary research to ensure effective landfill management and pollution prevention.

## Data Availability

The data that support the findings of this study are available from [Pervez Alam]. Still, restrictions apply to the availability of these data, which were used under license for the current study, and so are not publicly available. However, data are available from the authors upon reasonable request and with permission of [Pervez Alam].
